# Changes in treatment needs for chronic postoperative hypoparathyroidism during initiation of conventional treatment compared to stable phase of treatment

**DOI:** 10.1002/edm2.269

**Published:** 2021-06-01

**Authors:** Pernille Storm, Line Underbjerg, Lars Rejnmark

**Affiliations:** ^1^ Department of Endocrinology and Internal Medicine Aarhus University Hospital, Palle Juul‐Jensens Boulevard Aarhus Denmark; ^2^ Institute of Clinical Medicine Aarhus University Aarhus Denmark

**Keywords:** alfacalcidol, calcium, hypercalcaemia, hypocalcaemia, hypoparathyroidism, vitamin d

## Abstract

**Introduction:**

In our clinical experience, need for doses of active vitamin D and calcium supplements changes during the period following a diagnosis of postsurgical hypoparathyroidism (HypoPT), but only sparse data are available. In the present study, we aimed to investigate the magnitude of changes in need for activated vitamin D (alfacalcidol) and calcium supplements during initiation of therapy as well as time to be expected until a stable phase was achieved. Furthermore, we determined the frequency of (unexpected) episodes of hypo‐ and hypercalcaemia after reaching a steady state for alfacalcidol and calcium.

**Methods:**

Retrospective study of twenty‐four patients with chronic postsurgical HypoPT (>6 months) diagnosed from 2016 to 2018. Data were extracted from medical records on doses of alfacalcidol and calcium as well as ionized plasma calcium levels (P‐Ca^2+^) from time of diagnosis and until 86 weeks after surgery.

**Results:**

Patients were treated with alfacalcidol and calcium in order to maintain a stable concentration of P‐Ca^2+^. Our data demonstrated a great variation in treatment needs until 11 weeks after surgery, where the mean doses of alfacalcidol stabilize, while calcium doses stabilized a bit earlier. After the stable phase had emerged, 21 out of 24 patients continued to have one or more episodes of spontaneous hypo‐ or hypercalcaemia.

**Conclusions:**

Patients with chronic HypoPT attain a steady state for alfacalcidol 11 weeks after the diagnosis. Continuous monitoring of P‐Ca^2+^ is of continued importance after reaching steady state due to a high frequency of spontaneous hypo‐ or hypercalcaemia.

## Graphical abstract

BLURB FOR ETOCA retrospective review of medical records on 24 patients with chronic postoperative hypoparathyroidism lead to the finding of a steady state for treatment with activated vitamin D 11 weeks after the diagnosis.

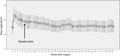

## 1 INTRODUCTION

Hypoparathyroidism (HypoPT) is a rare endocrine disorder characterized by hypocalcaemia caused by inadequate secretion of parathyroid hormone (PTH).^1^ Postsurgical HypoPT lasting less than 6 months is characterized as transient whereas chronic HypoPT has a duration of more than 6 months. Roughly, 75% of patients with HypoPT have acquired the disease due to anterior neck surgery where the parathyroid glands have been damaged or removed.^2^ A recent review reported a large variation in the incidence of HypoPT following surgery. The prevalence of transient HypoPT varied from 0 to 46% and chronic HypoPT from 0.9% to 4.4%.^3^

The purpose of treatment is to normalize plasma calcium levels in order to avoid hypo‐ and hypercalcaemia and the long‐term complications. The conventional treatment for HypoPT is supplements with active vitamin D (alfacalcidol or calcitriol) and oral calcium.^4^ The needs (doses) for active vitamin D in relation to acquiring HypoPT have not been well reported so far. Furthermore, only sparse data are available on how doses of active vitamin D need to be adjusted during the course of the disease.^5^, ^6^ One of the only available studies suggests that changes in calcium supplements and active vitamin D are only needed within the first month.^5^

This study aims to investigate the treatment needs for active vitamin D and calcium in the first period after surgery compared to treatment needs during stable phase of HypoPT, as we hypothesize that the patients attain a stable phase of treatment with calcium and active vitamin D during the first month after surgery.

## 2 MATERIAL AND METHODS

**FIGURE 1:**
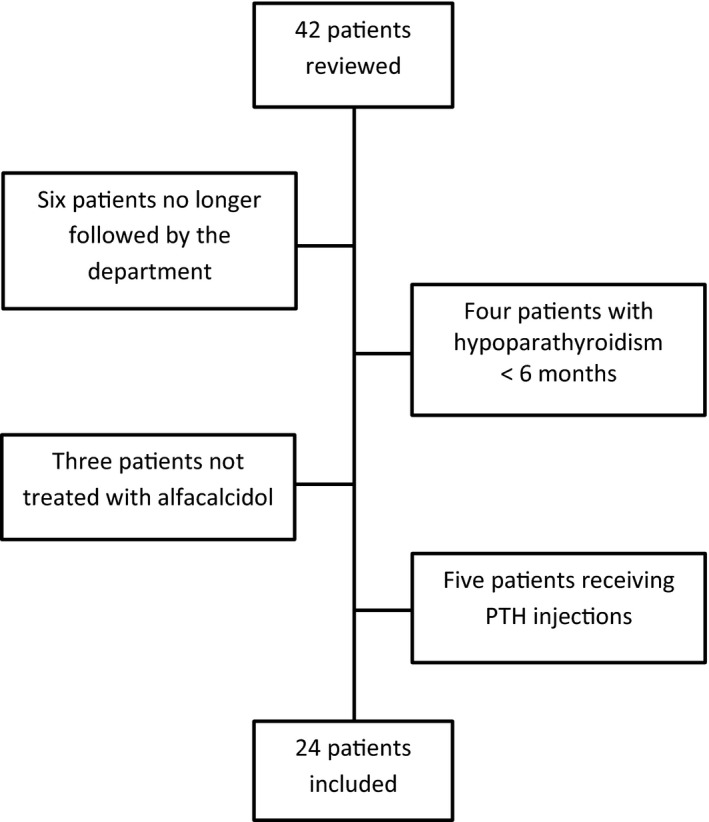
Flowchart showing included and excluded patients

We included 24 patients diagnosed with chronic HypoPT from 2016 to 2018 due to anterior neck surgery, who were treated with activated vitamin D and calcium postoperatively (Figure 1). Chronic HypoPT was defined as low P‐Ca^2+^ levels due to inappropriate low levels of circulating PTH lasting more than 6 months,^7^ necessitating treatment with activated vitamin D and calcium.

Patients with postsurgical HypoPT were identified by medical records at our Department of Endocrinology and Internal Medicine, Aarhus University Hospital, Aarhus, Denmark.

Data were extracted from medical records prior to and after surgery. The end of the follow‐up was 86 weeks after surgery, as data were available for all patients in this period. Patients who were no longer followed by our department were excluded (*n* = 6). Furthermore, patients were excluded if HypoPT lasted less than 6 months (*n* = 4), if not treated with alfacalcidol postoperatively (*n* = 3) or if they received PTH injections (*n* = 5) as this affects the required doses of alfacalcidol^4^ (Figure 1). As no examinations were performed as a part of a prospective study, the scientific committee classified the study as a register study without the need for approval. However, access to patients’ charts was granted by the Danish patient safety authority (#31‐1521–437) and the study was approved by the Danish Data Protection Agency (#2016‐051–000001).

### 2.1 Measurements

Medical records were reviewed with focus on P‐Ca^2+^ and changes in doses of calcium supplements and alfacalcidol. Patients diagnosed with postoperative hypocalcaemia were initially admitted to our department for monitoring and treatment of calcium homeostasis. After initial stabilization, patients were followed up at our out‐patient clinic for further titration with less frequent measurement of calcium levels on the discretion of the treating physician. After stabilization, calcium levels are measured routinely every 3^rd^ month, or more frequently, if needed (eg suspected hypo‐ or hypercalcaemia). If dose adjustments were needed, calcium levels were re‐measured 3–14 days later and the patient was followed closely until normocalcaemia was re‐established. If a patient was not seen for a given week, normocalcaemia was assumed as well as unchanged doses were assumed since the last clinical visit with last values carried forward. Calcium levels were measured in blood drawn in the morning and prior to medication. In the beginning of the disease, levels were sometimes measured several times a day. However, for the purpose of this study, we only used fasting morning samples. Ionized plasma calcium was measured immediately by an automated electrochemical method (Nova 8), and levels were adjusted to a pH value of 7.4. All patients were treated at our department according to the standard of care as detailed in the European guidelines for monitoring and treatment of chronic HypoPT, aiming at P‐Ca^2+^ levels in the lower half of the reference interval or slightly below the lower limit of normal.^7^ The reference for ionized calcium in our laboratory is 1.18–1.32 mmol/L. In the context of guidelines allowing for a target range for plasma calcium levels slightly below lower limit of normal, we defined hypocalcaemia as levels ≤1.15 mmol/L.

Intake of calcium supplements was reported as mg/week and alfacalcidol was reported as µg/week. Furthermore, baseline status (prior to surgery) of plasma levels of 25‐hydroxyvitamin D (25(OH)D), PTH and estimated glomerular filtration rate (eGFR) were retrieved.

Days with mild hypocalcaemia (P‐Ca^2+^<1.15 mmol/L) were counted for each patient, as number of days between two measurements showing P‐Ca^2+^ between 1.00 and 1.15 mmol/L.

Days with moderate to severe hypocalcaemia (P‐Ca^2+^<1.00 mmol/L) were counted for each patient as number of days between two measurements showing P‐Ca^2+^<1.00 mmol/L.

Days with hypercalcaemia were assessed in a similar manner by counting days with mild hypercalcaemia (P‐Ca^2+^ between 1.32 to 1.60 mmol/L) and days with moderate to severe hypercalcaemia (P‐Ca^2+^>1.60 mmol/L).^8^

### 2.2 Analyses

Determination of time when stable phase appeared was done by visual inspection of plots representing average doses of alfacalcidol and calcium. We measured weekly percentage change in alfacalcidol doses, where a stable phase was defined as changes less than ± 10% in requirement of doses for 80% of the patients.

We determined episodes with hypo‐ or hypercalcaemia showing the number of patients per week with respectively no, mild or moderate to severe hypocalcaemia, as well as hypercalcaemia.

### 2.3 Statistics

Data were tested for normal distribution using QQ‐plots. Data were reported as mean with standard deviation (SD) if the distribution was normal and as median with interquartile range (25%–75% percentiles) if data were not normally distributed. Fisher's exact test was used in order to test the difference before the stable phase of treatment compared to in the stable phase of treatment regarding events of hypo‐ and hypercalcaemia.

We performed all calculations using the IBM Statistical Package for Social Science (SPSS 24.0) for Windows (IBM Corp) and LibreOffice Calc (version 6.1.5.2) for Windows (DK).

## 3 RESULTS

24 patients were included in the study with a mean age of 54 years (from 21 to 72 years old). Table 1 summarizes characteristics prior to surgery, showing that 71% were women and 29% were men. Fifty percentage of the participants had anterior neck surgery due to thyroid cancer, 25% due to primary hyperparathyroidism (PHPT), 17% due to non‐toxic goitre, 4% due to Graves’ disease and 4% due to toxic goitre. Prior to the operation, the mean value for P‐Ca^2+^ was 1.33 mmol/L (Ref: 1.15–1.32 mmol/L) which was slightly above the normal range. This was attributable to hypercalcaemia among patients with PHPT, as the mean level of P‐Ca^2+^ before surgery for patients with PHPT was 1.43 mmol/L, whereas the mean level of P‐Ca^2+^ was 1.27 mmol/L when excluding patients with PHPT. A similar pattern was seen in PTH measurements, where the mean value for PTH measured in plasma (P‐PTH) for all patients was 6.9 pmol/L (Ref: 1.6–6.9 pmol/L). The mean value for P‐PTH in PHPT patients was 11.4 pmol/L, whereas patients without PHPT had a mean P‐PTH within the normal range at 4.9 pmol/L. BMI, 25(OH)D and eGFR were within a normal range.

### 3.1 Changes in treatment needs

**FIGURE 2:**
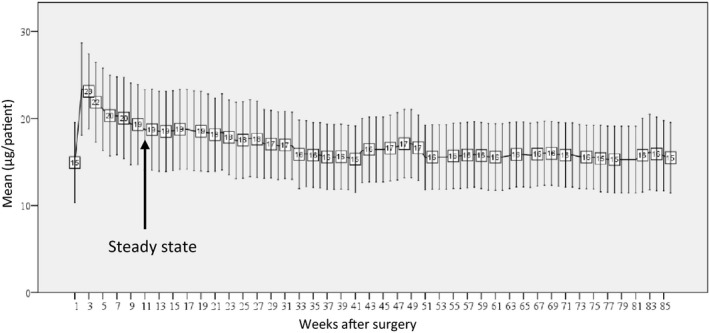
Mean (95% CI) dose of alfacalcidol (µg/week)

**FIGURE 3:**
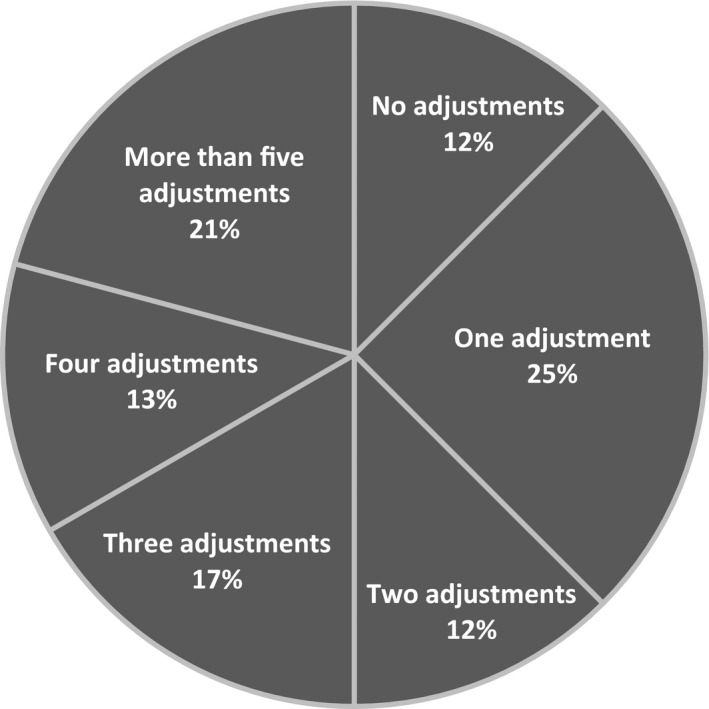
Percentages of patients needing adjustments in doses of alfacalcidol during stable phase of treatment

Mean doses of alfacalcidol per patient given per week are summarized on Figure 2. In the first weeks when HypoPT was diagnosed, the weekly mean dose of alfacalcidol increased to a maximum 3 weeks after surgery at 23 µg/patient/week, henceforward, the needed mean dose decreased to a stationary level 11 weeks after surgery with a dose of 19µg/patient/week. After week 11, the changes in treatment needs were sparse with a modest decrease to approximately 16µg/patient/week 33 weeks after surgery. After the stable phase had emerged, one patient did have a marked increase in needs for alfacalcidol doses week 40 to 50 which was of such magnitude that it affected the overall median levels. In addition to this patient, 20 other patients did have changes of less magnitude in their needs for alfacalcidol after they entered a stable phase of treatment. Six patients had just one adjustment of their alfacalcidol dose in the stable phase of treatment, three patients had two adjustments, four patients had three adjustments and eight patients had more than four adjustments of their treatment with alfacalcidol in the stable phase (Figure 3).

**FIGURE 4:**
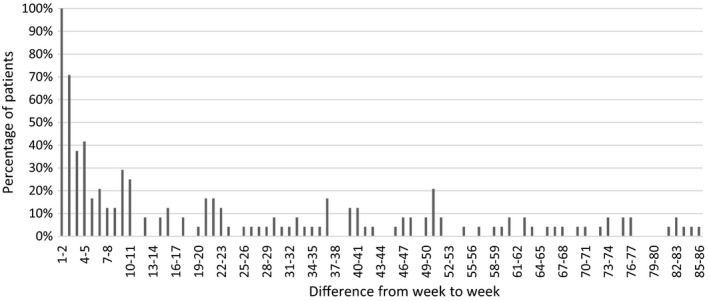
The percentage of patients with a weekly change of more than ± 10% in doses of alfacalcidol

Figure 4 demonstrates the relative number of patients with a weekly change in alfacalcidol needs of more than ± 10%. In the first weeks after surgery, the changes in treatment needs were more than ± 10% for most of the patients, as alfacalcidol doses changed more than ± 10% from week 1 to week 2 for all of the patients. From week 2 to 3, 70% had a change in alfacalcidol doses of more than ± 10%. Subsequently, less than 50% had a weekly change in alfacalcidol of more than ± 10%. Eleven weeks after surgery less than 20% of the patients had changes of more than ± 10% until week 51, which agrees with our interpretation of Figure 2, suggesting that 11 weeks after surgery the patients were relatively stable and remained stable for more than 3 months.

**FIGURE 5:**
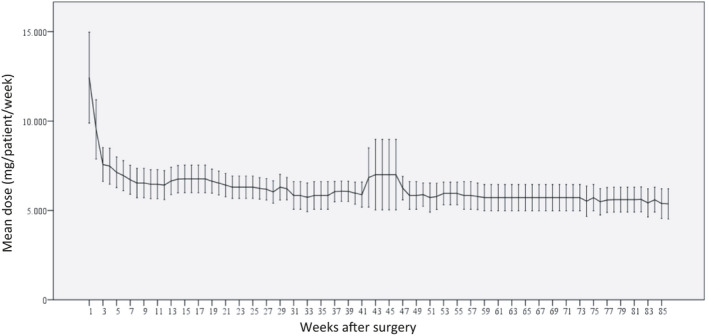
Mean (95% CI) dose of calcium (mg/week)

Treatment with calcium supplements is shown on Figure 5. Patients were given high doses in the first weeks after surgery, where the mean dose of calcium was approximately 12 g/patient/week. Subsequently, the mean dose of calcium decreased to around 6 g/patient/week 5–7 weeks after surgery and remained stable apart from 41 to 47 weeks after surgery where the mean dose increased. This increase in needs for calcium was due to the same patient, who had a marked increase in needs for alfacalcidol in the same period. Similar to alfacalcidol, the change in the needs for calcium supplements for this patient was of such magnitude that it affected the overall mean levels. When excluding this patient mean dose of calcium and alfacalcidol remained stable. No obvious reasons explain this marked increase in needs for calcium supplements and dose of alfacalcidol, except that this patient had experienced catarrhalis up to the date of the blood measurement. We can only speculate if this has caused an impaired gastrointestinal absorption necessitating increased needs.

Calculating the treatment needs for native vitamin D supplements showed similar findings to the needs described for both alfacalcidol and calcium supplements. Data not shown.

### 3.2 Measurement of serum calcium during treatment

The number of patients with hypocalcaemia, stratified by whether hypocalcaemia was mild or moderate to severe, are shown on Figure S1A. Moreover, the median number of days per week with hypocalcaemia is shown for patients suffering from hypocalcaemia. Therefore, for each week, the measurement does not include patients without hypocalcaemia. The number of patients with hypocalcaemia is displayed on the y‐axis on the left site, whereas days with hypocalcaemia are shown on the y‐axis on the right site.

In the first week after surgery, 23 patients (96%) suffered from hypocalcaemia with a median duration of five days. Five of the 23 patients (22%) had severe hypocalcaemia (P‐Ca <1.0 mmol/L). In the second week after surgery, 17 patients had hypocalcaemia with a median duration of 5 days. During the beginning of the treatment, all of the patients had episodes with hypocalcaemia whereas only 1–2 patients per week had episodes with hypocalcaemia after the stable phase occurred. Four patients had no episodes with hypocalcaemia after week 5, which means that the remaining 20 patients all had one or more episodes with hypocalcaemia after the stationary phase of treatment had occurred. Six patients had moderate to severe hypocalcaemia before the stable phase occurred, whereas only two patients had moderate to severe hypocalcaemia in the stable phase of treatment.

The most severe case of hypocalcaemia had P‐Ca^2+^ at 0.80 mmol/L, which was measured prior to the stable phase of treatment.

Figure S1B shows the number of patients per week who experienced either no hypercalcaemia, mild hypercalcaemia or moderate to severe hypercalcaemia, which can be read off of the y‐axis on the left site. Furthermore, the figure shows the median number of days per week that patients with hypercalcaemia suffered from hypercalcaemia which can be read off of the y‐axis on the right site.

Nine patients (38%) had one or more episodes with hypercalcaemia prior to the stable phase of treatment, while 11 patients had episodes with hypercalcaemia during the stable phase (*p *= .56). The episodes with hypercalcaemia were sporadic and randomly occurring. Moreover, the median duration of the episodes varied from 1 to 7 days/week. As it is seen on Figure S1B, there was no difference between the stable phase of treatment and prior to the stable phase regarding the length of the episodes with hypercalcaemia or the number of patients experiencing hypercalcaemia. The most severe case of hypercalcaemia had P‐Ca^2+^ at 1.91mmol/L.

## 4 DISCUSSION

Patients were treated with alfacalcidol and calcium supplements as recommended by guidelines^7^, ^9^ in order to obtain and maintain a stable concentration of P‐Ca^2+^ in the lower part of the reference range. On a contrary to other studies, our data demonstrated that treatment needs varied greatly until 11 weeks after surgery, where the mean doses of alfacalcidol became more stable, which occurred a bit earlier for doses of calcium. The stable phase occurred much later than former studies.^5^ Days with hypocalcaemia occurred randomly and were not only in the beginning of the treatment but also in the stable phase of treatment, although the number of patients experiencing hypocalcaemia decreased concurrently with the number of weeks after surgery.

The gender distribution seen in our study with 29% men and 71% women was in consistency with other studies investigating postsurgical hypoparathyroidism.^10^, ^11^

A major observation made in this study was that we could expect a steady state for doses of alfacalcidol approximately 11 weeks after the operation causing HypoPT. Reasons for the increased treatment needs at the beginning needs further investigations. Vitamin D is a fat‐soluble vitamin, and we can only speculate that there is a need for the body to become saturated with vitamin D before a new steady state is achieved.

Only a few prior studies have reported on doses of alfacalcidol needed during initiation of treatment for HypoPT. In a study by Halabe et al,^5^ 17 patients were followed daily for the first two weeks, then every 2 weeks for the first month, then monthly for the next 3 months and every 3–6 months for the rest of the 10 years follow‐up. The study reported that a significant effect of alfacalcidol on P‐Ca^2+^ levels was achieved as early as during the first week of treatment and remained in a stable level during follow‐up. This differs from our data suggesting that a stable level was not achieved prior to 11 weeks of treatment. An explanation could be the difference in the study populations as 15 patients had postsurgical HypoPT and 2 patients non‐surgical HypoPT,^5^ whereas our study only included patients with chronic postsurgical HypoPT. Moreover, the indication for surgery was not specified in the study by Halabe et al,^5^ and finally, the patients were hospitalized the first two weeks after diagnosed with HypoPT. In this period, they had a daily diet containing strict amounts of protein, fat, carbohydrate, calcium, phosphorus and sodium, which was not the case for the patients in our study. It would be of interest to investigate the possibility of obtaining a steady state sooner with intensification of the initial treatment with hospitalization and diet standardization, as seen by Halabe et al.^5^

The mean maintenance dose of alfacalcidol in the study by Halabe et al^5^ was 1 µg/day with a range of 0.5–2.5 µg/day. This was a much lower dose than the dose needed for patients in our study, that is 16 µg/week at 33 weeks after surgery. However, the needed amount of calcium in the first weeks of treatment (7–13 g/week in week 1–11) and at the end of this study (6 g/week) was similar to Halabe et al,^5^ where the needed dose of calcium was 1.5–2.0 g/day in the beginning of treatment and 0.5–1.0 g/day as maintenance dose.

The population in this study is not quite comparable to the population in Halabe et al,^5^ due to the difference in treatment of HypoPT. Treatment of HypoPT with alfacalcidol and calcium supplements can be titrated differently. At our department, patients are treated with relatively high doses of alfacalcidol in combination with lower doses of calcium whereas other institutions are treating HypoPT with high doses of calcium and relatively lower doses of activated vitamin D. These differences in the titration of the treatment are of course of importance as it influences on the needed doses. It would be interesting to have a direct comparison of the different regimes. It seems that doses of calcium supplements stabilized earlier than doses of alfacalcidol. Dose adjustments for calcium and alfacalcidol were based on clinical judgement. Further prospective studies using a predefined treatment protocol are needed to define the best treatment regimens during initiation of treatment.

A characteristic among HypoPT patients seen in this study was the sudden changes in treatment needs due to fluctuations in calcium levels in the blood, which also is well known from other studies suggesting biochemistry to be monitored at regular intervals (every 3–6 months) and weekly after changes in treatment.^7^, ^10^ It is poorly understood how some of the symptoms and long‐term complications among HypoPT patients occur, but fluctuations in calcium and phosphate levels are presumably associated with an increased risk of long‐term complications.^12^ Increased levels of calcium and calcium‐phosphate product in the blood are associated with impaired renal function, nephrocalcinosis, nephrolithiasis and renal failure.^13^, ^14^, ^15^ Moreover, a recent review by Mannstadt et al^16^ shows that conventional treatment increases the risk of hypercalciuria due to increased excretion of calcium in the urine caused by the lack of PTH‐induced reabsorption of calcium in the kidneys. This study was from the United States where high doses of calcium are used relative to active vitamin D, which might explain the higher risk of hypercalciuria.^15^

More studies are needed to investigate why sudden variations in the needed treatment occur. Replacement therapy with full‐length (1–84) recombinant human (rh) PTH has been available for treatment of HypoPT in the United States and the European Union for the last couple of years and evidence have shown it to be useful especially for those who require high doses of calcium and alfacalcidol.^4^, ^7^ It would be of relevance to investigate whether patients treated with the missing hormone (rh‐PTH) or patients with an (insufficient) residual function of PTH have less variations in P‐Ca and thereby more stable needs of treatment.

Hypercalcaemia is not only associated with increased risk of renal diseases, but also with an increased mortality as well as high phosphate levels increase the risk of infections.^12^ The risk of cardiovascular diseases increase when suffering from HypoPT for more than 20 years, when having more than 4 episodes of hypercalcaemia and when patients have adequately low levels of calcium corresponding to hypocalcaemia.^12^

Therefore, it is relevant to investigate conventional treatment in order to determine whether the treatment needs change during the time patients suffer from HypoPT in order to reduce the long‐term effects of inadequately treated HypoPT.

A major strength of this study is the systematic assessment of treatment needs in terms of active vitamin D and calcium supplements in a group of patients recently diagnosed with HypoPT and followed for more than one year after the diagnosis. To the best of our knowledge, no prior studies have reported this. However, our study has several limitations. First, we only included a relatively small group of patients. However, HypoPT is a rare disease, hence it is difficult to make studies with a larger group of patients. Moreover, this is a retrospective study based on patient records, which is better than if depending on patient's memory due to recall bias. Nevertheless, a prospective design would have been better in order to investigate the reasons for sudden development of hypo‐ or hypercalcaemia, which often are unknown and therefore impossible to explore in a retrospective design. Moreover, a prospective design could have included measurements of plasma phosphate and urine calcium in order to investigate how the treatment influence on these. A final limitation is that this study only included patients suffering from chronic HypoPT, which means that patients suffering from HypoPT less than 6 months characterized as transient were excluded. It is unknown whether patients who reestablish their parathyroid function during the first 6 months have more or less fluctuations in P‐Ca^2+^ levels than patients with permanent HypoPT.

In conclusion, a steady state for doses of alfacalcidol occurred approximately 11 weeks after surgery. However, the patients continued to have unexpected episodes of hypo‐ and hypercalcaemia after becoming stable, which means that it is important to continue monitoring patients with chronic HypoPT after stabilization of the treatment. Hence, we reject our hypothesis that the patients attain a stable phase of treatment with calcium and active vitamin D as early as during the first month after surgery.

## CONFLICTS OF INTEREST

The authors declare that they have no conflicts of interest.

5

**TABLE 1:** Characteristics prior to surgery for all included patients (*N* = 24)

Patients	Reference range	All
Gender, *n* (%)		
Male		7 (29)
Female		17 (71)
Age (years), mean (min‐max)		54 (21‐72)
Reasons for surgery, *n* (%)		
Thyroid cancer		12 (50)
PHPT		6 (25)
Non‐toxic goitre		4 (17)
Graves’ disease		1 (4)
Toxic goitre		1 (4)
Perioperative values, mean (*SD*)		
Calcium, ionized (mmol/L)	1.18‐1.32	1.33 (0.1)
PHPT		1.43 (0.1)
Non‐PHPT		1.27 (0.1)
PTH (pmol/L)	1.6‐6.9	6.9 (3.9)
PHPT		11.4 (3.8)
Non‐PHPT		4.9 (1.8)
25‐hydroxy Vitamin D (nmol/L)	50‐160	72.8 (27.9)
eGFR (ml/min)	> 60	78.8 (16.6)
BMI (kg/m^2^)	18.5‐25	25.1 (5.0)

Note

Mean with standard deviation (*SD*) or number (percentages).

Abbreviation: PHPT: Primary hyperparathyroidism, Non‐PHPT: Non‐primary hyperparathyroidism.

## Supporting information

Fig S1

## Data Availability

The data that support the findings of this study are available from the corresponding author upon reasonable request.
