# Supplementary material for: Changes in treatment needs for chronic postoperative hypoparathyroidism during initiation of conventional treatment compared to stable phase of treatment
Source: Endocrinol Diabetes Metab. 2021 Jun 1;4(3):e00269. doi: 10.1002/edm2.269 (PMC8279598; doi:10.1002/edm2.269)
Supplement: Supplementary file 1 — Fig S1 [file EDM2-4-e00269-s001.docx]

**Supplementary figure**

Figure text: Patients with normal P-Ca^2+^ were excluded from the measurement of median number of days with hypo- and hypercalcemia.
